# Lactate-induced autophagy activation: unraveling the therapeutic impact of high-intensity interval training on insulin resistance in type 2 diabetic rats

**DOI:** 10.1038/s41598-023-50589-0

**Published:** 2024-01-11

**Authors:** Hossein Pirani, Afsaneh Soltany, Maryam Hossein Rezaei, Adeleh Khodabakhshi Fard, Rohollah Nikooie, Kimya Khoramipoor, Karim Chamari, Kayvan Khoramipour

**Affiliations:** 1 Department of Basic Sciences, Chabahar Maritime University, Chabahar, Iran; 2 Department of Biology, Faculty of Science, University of Shiraz, Shiraz, Iran; 3 Department of Exercise Physiology, Faculty of Physical Education and Sport Sciences, Shahid Bahonar University, Kerman, Iran; 4 Department on Nutrition, Faculty of Public Health, Kerman University of Medical Sciences, Kerman, Iran; 5 Neuroscience Research Center, Institute of Neuropharmacology, Kerman University of Medical Sciences, Kerman, Iran; 6 Department of Nursing, Faculty of Nursing and Midwifery, Kurdistan University of Medical Sciences, Kurdistan, Iran; 7 Higher Institute of Sport and Physical Education, ISSEP Ksar Said, Manouba University, Manouba, Tunisia; 8 Neuroscience Research Center, Institute of Neuropharmacology and Department of Physiology and Pharmacology, Afzalipour School of Medicine, Kerman University of Medical Sciences, Kerman, Iran

**Keywords:** Cell biology, Physiology

## Abstract

Impaired autophagy is a hallmark of diabetes. The current study proposed to investigate if high intensity interval training (HIIT) induced lactate accumulation could stimulate autophagy in type 2 diabetic male rats. 28 male Wistar rats were randomly assigned into four groups: Healthy Control (CO), Diabetes Control (T2D), Exercise (EX), and Diabetes + Exercise (T2D + EX). Diabetes was induced by feeding high-fat diet and administrating single dose of streptozotocin (35 mg/kg). After becoming diabetic, the animals in the exercise groups (EX and T2D + EX) performed an eight-week HIIT (4–10 interval, 80–100% Vmax, 5 days per week). Serum levels of lactate, glucose and insulin as well as the levels of lactate, pyruvate, lactate transporter monocarboxylate transporter 1 (MCT1), phosphorylated mitogen-activated protein kinases (p-MAP 1 and 2), phosphorylated extracellular signal-regulated protein kinases 1 and 2 (p-ERK 1 and 2), mammalian target of rapamycin (p-mTOR), ribosomal protein S6 kinase beta-1 (p-70S6k), p90 ribosomal S6 kinases (p-90RSK), autophagy related 7 (ATG7), Beclin-1, microtubule-associated protein 1A/1B, and 2A/2B -light chain 3 levels (LC3-I), (LC3- II), (LC3I/LC3II) in soleus muscle were measured. Homeostatic Model Assessment for Insulin Resistance (HOMA-IR) and serum glucose was lower in T2D + EX compared to T2D group (*P* < 0.0001). While serum and soleus muscle levels of lactate was not different between T2D and T2D + Ex, the levels of Pyruvate (*P* < 0.01), MCT1, p-ERK1/2, p-mTOR, p70S6k, P-90RSK, ATG7, LC3-II, and LC3-II/LC3I ratios were higher in T2D + EX compared to T2D group (*P* < 0.0001). We concluded that eight weeks of high-intensity interval training could activated ERK/P90SRK while inhibiting mTOR/P70S6K signaling pathway in lactate dependent manner. It means increased autophagy which resulted in improve insulin resistance (IR) and reduce blood glucose.

## Introduction

Diabetic mellitus (DM), a chronic, metabolic disease, is currently considered the seventh leading cause of death worldwide^1,2^. In addition to causing many other diseases such as cardiovascular failure, neuropathy, nephropathy and retinopathy, DM has imposed huge medical costs on the health systems. It has been reported that 214.8 million dollars are spent annually on the outpatient treatment of DM and another 1.45 billion dollars on hospitalized DM patients. Taking into account other disorders caused by DM, the medical expenses of DM exceed 100 billion dollars annually^3^. These have made DM as one the top priority for countries worldwide^4^.

The first step in managing DM is to uncover the pathophysiology of this disease in order to propose preventive and therapeutic solutions. Impaired autophagy is a leading pathophysiological mechanism of DM^5^. Autophagy is a cellular process that degrades senescent or damaged organelles and proteins^6^. It can be selective or nonselective. Under stress and starvation, nonselective autophagy increases helping with hydrolyzing cytosolic components to provide cell nutrients. On the other hand, selective autophagy defined as specific targeting of damaged organelles or invading pathogens^7^.

It has been shown that IR (insulin resistance) could disrupt autophagy^8^. Several animal studies showed negative correlation between IR and muscle autophagy but one study reported the positive correlation^9^. Furthermore Møller et al. showed that the expression of autophagy-related genes (ATGs) (e.g. SQSTM1/p62 and ATG14) and proteins (e.g. LC3-II and ATG5) decreased in skeletal muscle of patients with type 2 DM^10^. Several mechanisms regulate autophagy in skeletal muscle. For example, activation of the AKT/mTORC1/PI3K pathway inhibits autophagy^11^. On the other hand, AMPK activates several ATGs, including LC3-II and Gabarapl1, which are genes that increase phagosome formation^12^.

Targeting autophagy has suggested as a therapeutic approach for DM. Recently Nikooei et al.,^13^ reported that lactate could regulate autophagy in skeletal muscle by activating ROS-mediated ERK1/2/mTOR/p70S6K pathway. The oxidation of lactate to pyruvate is associated with the production of NADH, which alters cytosolic redox potential and increases the production of ROS in mitochondria. This increased ROS levels can activate the ERK1/2/mTOR/p70S6K signaling pathway, thereby increasing autophagy in skeletal muscle^13,14^.

Exercise is considered as the main stimulator of lactate production in our body^15^. Because of severity, high intensity interval training (HIIT) stress anaerobic more than aerobic metabolism leading to more lactate accumulation^16^. Therefore, we hypothesized that HIIT could attenuate T2D induced autophagy impairment through lactate dependent activation of ERK1/2/mTOR/p70S6K signaling pathway. The objective of this study was to explore the impact of an 8-week HIIT on autophagy in male rats with T2D, with a particular emphasis on lactate levels in the soleus muscle. The soleus muscle, characterized by its slow-twitch properties and possession of a lactate transporter, was a focal point in our investigation.

## Materials and methods

### Animals

Male Wistar rats (average age: 8 weeks), weighing 200–250 g were purchased from the Kerman University of Medical Sciences animal farm. The animals were housed in a 12-h light/dark cycle at constant temperature (22 ± 1/4 °C), and humidity (50 ± 4%) while had ad libitum access to food and water. After one week of adaptation, animals were randomly divided into four groups (n = 7 in each group) as control (CO), type 2 diabetes (T2D), exercise (EX) and diabetes + exercise (T2D + EX). All procedures were carried out with the approval of the Kerman University of Medical Sciences Experimental Animals Ethics Committee (ethical code: IR.KMU.ERC.1399.688). All experiments were performed in accordance with the American Veterinary Medical Association and the Declaration of Helsinki.

### Induction of diabetes

The animals in T2D and T2D + EX were fed a high-fat diet (HFD) (60% fat, 20% carbohydrate and 20% protein) for 2 months. After that, animals were injected with low-dose (35 mg/kg) streptozocin (STZ) which was dissolved in dissolved in a 0.1 mM citrate–phosphate buffer (pH4.5) 3 days after the injection, blood glucose levels were measured with a glucometer. Animals with blood glucose levels of 300 mg/dl or higher were accepted as diabetics^17,18^.

### Treadmill running protocol

Animals in training groups (EX, T2D + EX), were acclimated to treadmill running for 5 days (speed 8 m/min for 10 min). To determine the maximum speed (Vmax), rats first walk on the treadmill (6 m/min, 2 min, incline 0). The treadmill speed was then increased stepwise (every 2 min by 2 m/min) until exhaustion. The final effort of each rat was taken as Vmax. It should be noted that the maximum speed of the rats was re-measured every two weeks. The main training protocol is described in details in our previous studies^17,19–23^. Briefly, the HIIT protocol consisted of 4–10 intervals (2 min high intensity and 1 min low intensity interval) with 80–100% of Vmax.

### Serum and issue sampling

48 h after the last training session, all trained animals were anesthetized by intraperitoneal administration of ketamine (80 mg/kg) and xylazine (10 mg/kg). Blood samples were taken from the animal’s heart after 12 h of fasting. Then, the soleus muscles were carefully removed, immediately frozen in liquid nitrogen, and then stored at 80°C further analysis.

### Western blotting

The western blot method was used to examine the concentrations of ERK1/2, mTOR, P90RSK, p70S6K, MCT1, LC3-I, LC3-II, P62, Beclin1 and ATG7 in the soleus muscle. Western blot protocol is described in details elsewhere. Briefly, to lyse tissues, lysis buffer (150 mM sodium chloride, Triton X-100: 1%, 0.25% sodium deoxycholate, 0.1% SDS, 50 mM Tris, pH 8.0) was used. The samples were centrifuged at 12,000 rpm at 4 °C for 10 min, using an Eppendorf 5415R and the supernatant was collected. Samples were stored at − 80 °C before use, and the protein concentrations were determined using a Bradford assay. The proteins were separated by SDS-PAGE and transferred to a PVDF membrane for 1.5 h at 120 mv. Membranes were blocked with 5% skim milk for 1 h at the room temperature, and then incubated with specific primary antibodies for 18 h (ATG7 (B-9): sc-376212, BECN1 (E-8): sc-48341, , LC3-I/ LC3-II: # 2775, p70 S6 kinase α (C-18): sc-230, p-ERK 1/2 (Thr 177)-R: sc-16981-R, p-mTOR (Ser 2481 55.42): sc-293089, p-p62 (Thr 180/Tyr 182)-R: sc-17852-R, MCT1 (SLC16A1): #AMT-011, P- P90RSK (Thr 359/ Ser 363)-R: # 9364. After that, the membranes were washed with TBS-T and incubated with secondary antibodies conjugated with horseradish peroxidase. The proteins of interest were visualized using ECL (enhanced chemiluminescence) reagents. Finally, qualitative measurements were converted into quantitative data by the ImageJ software.

### Elisa

Lactate and pyruvate in serum and soleus muscle and insulin concentration levels were measured by ELISA technique (Rat ELISA Kit, Eastbiopharm, Beijing, China) according to manufacturer’s instructions^24,25^.

### Calculation of insulin resistance

The homeostasis model assessment (HOMA) was used to assess insulin resistance (HOMA-IR). HOMA-IR scores were calculated using the following formula: HOMA-IR = [(fasting glucose (mmol/L) × fasting insulin (µU/mL))/22.5]^26^.

### Statistical analysis

Results are presented as the mean ± standard error of the mean (SEM). Data normality was first tested with Kolmogorov Smirnov test. As the data was normal, we used Two Way ANOVA and Tukey post doc test to compare the variables between groups. The statistical significancy was set at 0.05. All statistical analysis performed by GraphPad Prism version 9.0

### Ethics approval

All procedures were carried out with the approval of the Kerman University of Medical Sciences Experimental Animals Ethics Committee (ethical code: IR.KMU.ERC.1399.688).

## Results

The study is reported in accordance with ARRIVE guidelines.

### Body weight, fasting blood glucose (FBG), serum insulin level and HOMA-IR

**Figure 1:**
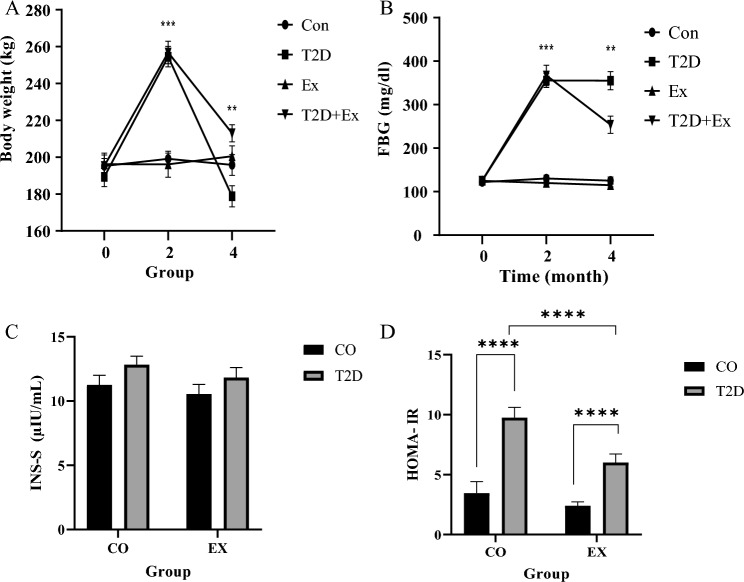
Body weight (**A**), FBG (**B**), INS-S (**C**), HOMA-IR (**D**). *FBG* fasting blood glucose, *INS-S* insulin level in serum, *HOMA-IR* homeostatic model assessment for insulin resistance. *CO* control, *T2D* type 2 diabetes, *EX* exercise. ****(*P* < 0.0001), ***(*P* < 0.001), **(*P* < 0.01) (n = 7 in each group).

Animals’ weight showed a significant increase in T2D and T2D + Ex groups after diabetes induction (2 months of high fat diet and STZ injection) (*P* < 0.001). In addition, the weight was decreased in T2D and T2D + Ex groups (*P* < 0.001) with more decrease in the T2D group (*P* < 0.01) (Fig. 1A). We assessed FBG to confirm our diabetes induction method. Our results showed that FBG was significantly increased after diabetes induction (2 months of high fat diet and STZ injection) (month 2) compared with baseline (month 0) in T2D and T2D + Ex group (*P* < 0.001), with no significant difference between these groups. In addition, HIIT reduced blood glucose significantly (*P* < 0.01) (Fig. 1B).

Serum insulin (INS-S) level showed no significant effect for T2D and Ex as well as their interaction (*P* > 0.05) (Fig. 1C). However, T2D had higher HOMA-IR than CO group (T2D effect) (f_1, 20_ = 23.80, *P* < 0.0001). No significant difference was found between EX and CO groups (EX effect). In addition, a significant difference was shown between T2D and T2D + EX groups (interaction) (f_1, 20_ = 24.60, *P* < 0.0001) (Fig. 1D).

### Lactate levels in serum and soleus muscle

**Figure 2:**
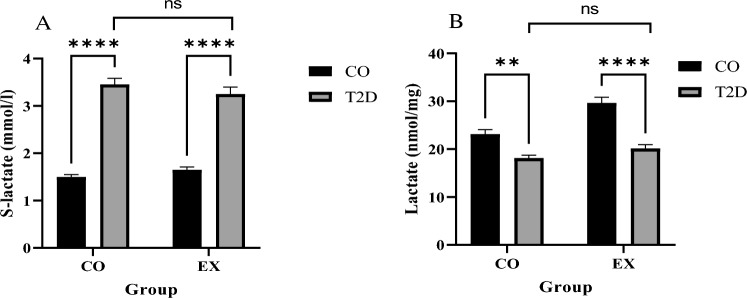
S-lactate: serum lactate levels (**A**), lactate levels in the Soleus muscle (**B**). *CO* control, *T2D* type 2 diabetes, *EX* exercise. *** (*P* < 0.001), ** (*P* < 0.01), ns (no significant difference), (n = 7 in each group).

Our results showed that serum lactate levels were significantly higher in T2D compared with CO (T2D effect), (f_1, 20_ = 281.2, *P* < 0.0001) but we saw no difference between CO and EX (EX effect) (f_1, 20_ = 0.055, *P* = 0.8), T2D + EX and T2D (interaction) (f_1, 20_ = 281.2, *P* = 0.78). Lactate levels in the Soleus muscle showed a significant difference between CO and T2D group (T2D effect) (f_1, 20_ = 56.47, *P* < 0.0001). EX group showed higher levels than CO group (EX effect) (f_1, 20_ = 19.40, *P* < 0.0003). But there was no significant difference between T2D and T2D + EX (interaction effect) (Fig. 2).

### MCT1

**Figure 3:**
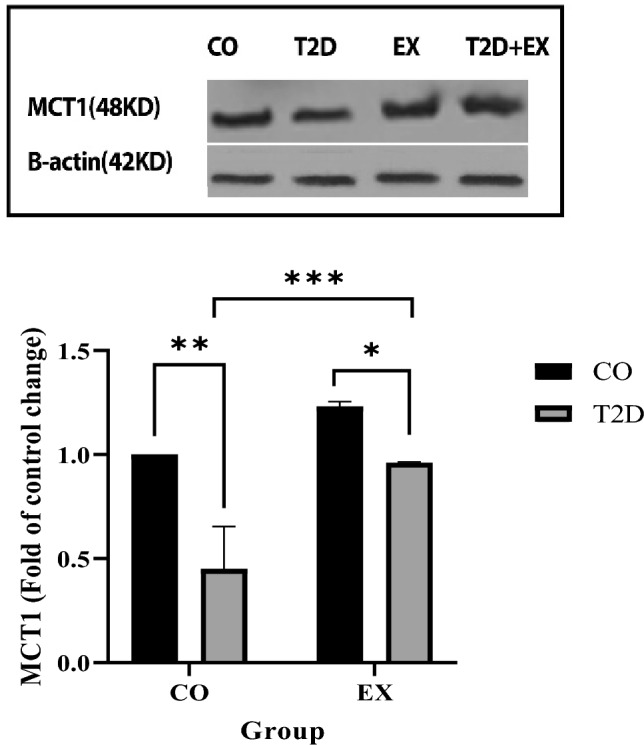
MCT1: lactate transporter monocarboxylate transporter 1. *CO* control, *T2D* type 2 diabetes, *EX* exercise. ****(*P* < 0.0001), (n = 7 in each group).

EX and T2D group showed higher (EX effect) (f_1, 20_ = 539.2, *P* < 0.0001) and lower (T2D effect) (f_1, 20_ = 662.0, *P* < 0.0001) MCT1 content in soleus muscle and showed lower levels than CO group. In addition, T2D + EX showed higher levels compared to T2D group (interaction) (f_1, 20_ = 77.19, *P* < 0.0001) (Fig. 3).

### Muscle pyruvate levels

**Figure 4:**
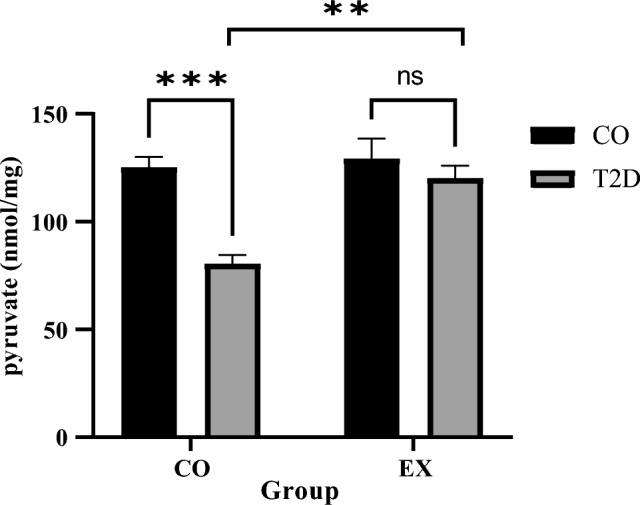
Pyruvate levels. *CO* control, T2D: Type 2 diabetes, EX: exercise. ***(*P* < 0.001), **(*P* < 0.01), ns (No significant difference), (n = 7 in each group).

Pyruvate levels were significantly lower in T2D compared to CO group (T2D effect) (f_1, 20_ = 16.85, *P* < 0.0006). No changes in pyruvate levels were seen between CO and EX group (EX effect). But we showed significant difference between T2D + EX and T2D groups (interaction effect), (f_1, 20_ = 7.446, *P* < 0.001) (Fig. 4).

### Autophagy related proteins

**Figure 5:**
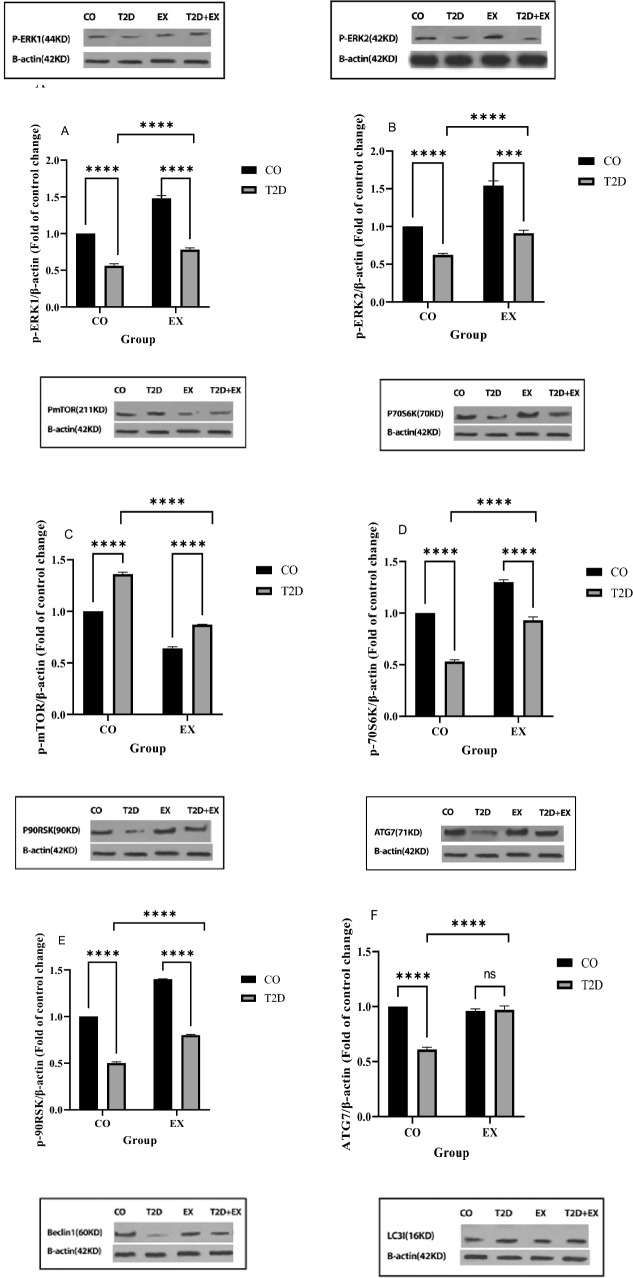

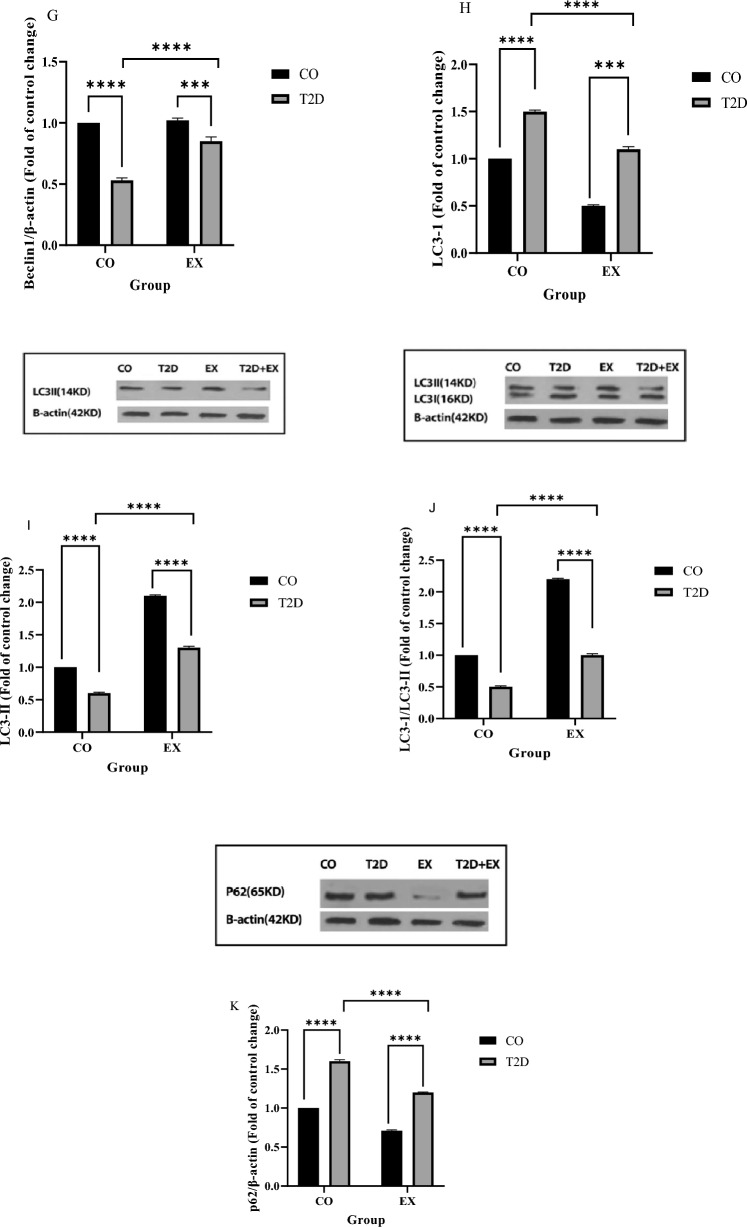
P- ERK1/2: phosphorylated mitogen-activated protein kinases (MAPKs) ½ (**A**,**B**), p- mTOR: mammalian target of rapamycin (**C**), p70S6k: Ribosomal protein S6 kinase beta-1 (**D**), P-90RSK: p90 ribosomal S6 kinases (**E**), ATG7: autophagy related (**F**), Beclin-1 (**G**), LC3-I (**H**), LC3-II (**I**), LC3I/LC3II (**J**), P62 (**K**). *CO* control, *T2D* type 2 diabetes, *EX* exercise. **** (*P* < 0.0001), *ns* no significant difference, (n = 7 in each group).

Our results showed that p-ERK1 (f_1, 20_ = 23.96, *P* < 0.0001) and p-ERK2 (f_1, 20_ = 11.49, *P* < 0.0002) levels showed a significant difference between T2D and T2D + EX (interaction effect). EX group showed lower (EX effect) p-ERK1 (f_1, 20_ = 173.6, *P* < 0.0001) and p-ERK2 (f_1, 20_ = 112.0, *P* < 0.0001) and T2D showed higher (T2D effect) p-ERK1 (f_1, 20_ = 460.6, *P* < 0.0001) and p-ERK2 (f_1, 20_ = 166.7, *P* < 0.0001) levels compared to CO group (Fig. 5 A, B). p- mTOR levels significantly decreased in EX (f_1, 20_ = 110.2, *P* < 0.0001, EX effect) and increased T2D (f_1, 20_ = 531.0, *P* < 0.0001, diabetes effect) compared to CO group in muscle. So, we showed a significant difference between T2D + EX and T2D (f _1, 20_ = 251.78, *P* < 0.0001, interaction effect) (Fig. 5C). As shown Fig. 5D, the level of p70S6k was lower in the T2D (T2D effect) (f_1, 20_ = 110.2, P < 0.0001) and higher in EX (EX effect) (f_1, 20_ = 110.2, P < 0.0001) compared to CO groups. In addition, we showed higher level of p70S6k in the T2D + EX compared to T2D group (interaction) (f_1, 20_ = 110.2, P < 0.0001). Exercise (f_1, 20_ = 16.07, P < 0.0001) and T2D (f_1, 20_ = 39.69, P < 0.0001) increase and decrease, p-p90RSK levels in the soleus muscle respectively. Furthermore, p-p90RSK levels were significantly higher in the T2D + Ex than T2D group (interaction effect) (f_1, 20_ = 32.80, P < 0.0001) (Fig. 5E). ATG7 levels were significantly lower in T2D with CO (T2D effect) (f_1, 20_ = 66.93, P < 0.0001). But ATG7 levels did not differ between EX with CO (EX effect). There were higher ATG7 levels in T2D + EX compared to T2D (interaction) (f_1, 20_ = 77.49, P < 0.0001) (Fig. 5F). Our results showed that Beclin-1 levels showed a significant difference between CO and T2D group (T2D effect) (f_1, 20_ = 198.4, P < 0.0001). T2D and T2D + EX showed higher levels than CO group (interaction effect) (f_1, 20_ = 43.59, P < 0.0001). But not significant difference was seen between EX and CO (EX effect) (Fig. 5G). Our results showed that LC3-I levels were higher in T2D (Fig. 5H), in addition LC3-II showed higher level in EX (Fig. 5I) and LC3-II / LC3-I increased and decreased in EX (f_1, 20_ = 211.5, P < 0.0001) and T2D (f_1, 20_ = 244.1, P < 0.0001) groups compared with Co group, respectively. In addition, this ratio was lower in T2D compared to T2D + Ex group (f_1, 20_ = 990.3, P < 0.0001) (Fig. 5J). P62 levels was decreased by EX (f_1, 20_ = 190.3, P < 0.0001) and increased by T2D (f_1, 20_ = 156.9, P < 0.0001). The T2D and EX interaction was also significant for P62 (f_1, 20_ = 275.4, P < 0.0001) (Fig. 5K).

## Discussion

Skeletal muscles account for 30–40% of total body mass and they are responsible for 80% of insulin-dependent glucose uptake. IR of skeletal muscle could lead to T2D^27^. A growing body of evidence have shown that autophagy could regulate skeletal muscle metabolism helping with improving IR^28^. Therefore, we aimed to study the effect of 8 weeks HIIT on autophagy in the soleus muscle of T2D rats with special focus on lactate. Our data showed that lactate mediates the increased autophagy induced by HIIT in the soleus muscle, which reduced IR and serum levels of glucose. Thus, our hypothesis has been approved.

The results showed that the Beclin1 level, one of the key regulatory factors of autophagy, was significantly lower in the T2D + EX group compared to the T2D group after 8 weeks HIIT. This data is in line with the result of Yang et al.,^4^ who reported increased muscular levels of Beclin1 after 8 weeks of swimming (60 min every day). Furthermore, the levels of Beclin1 in the soleus muscle decreased with 35 weeks of high-fat diet but increased after 10 weeks of running on treadmill^11^. Another important autophagy related protein is ATG7. Lu and colleagues^29^ confirmed the negative correlation between ATG7 and IR and reported that lower ATG7 levels are associated with an increased risk of diabetes. In our study, ATG7 levels showed a significant decrease in the T2D group, but increased after 8 weeks of HIIT. According to a report, the administration of an HFD for 16 weeks resulted in a decrease in the expression of ATG 7^30^. However, an increase in ATG7 was observed in skeletal muscle following an 8-week endurance^31^ and resistance training^32^. These findings are consistent with the results of our study. ATG7 plays a role in the conjugation of LC3-I during autophagy, leading to the formation of LC3-II. Increased ratio of LC3-II/LC3-I is necessary for autophagosome formation and considered as a hallmark of autophagy^33,34^. Our results showed that soleus muscle LC3-II levels decreased significantly in the T2D group compared to the CO group, and the T2D + EX group showed a significant higher levels of LC3-II. In addition, we saw an increase in LC3-1 levels in diabetic rats which could be due to a decreasing ATG7 level and also a lack of LC3-II lipidation. On the other hand, a decrease in LC3-1 levels after exercise can confirm its conversion to LC3-II. It has been reported that the ratio of LC3-II/LC3-1 decreased in liver of diabetic mice, indicating the reduction in autophagy^35^. Furthermore, Jamart et al.^36^ demonstrated that LC3-II levels increased after running on a treadmill human skeletal muscle. In contrast to our findings, resistance training resulted in a decrease in the LC3-II/LC3-I ratio^32^. Furthermore, LC3-I increased in human skeletal muscle whereas there was no difference in LC3II after 8-week resistance training^37^. These inconsistencies could be attributed to differences in the type of exercise (HIIT vs resistance training) and the type of subjects (rats vs human).

**Figure 6:**
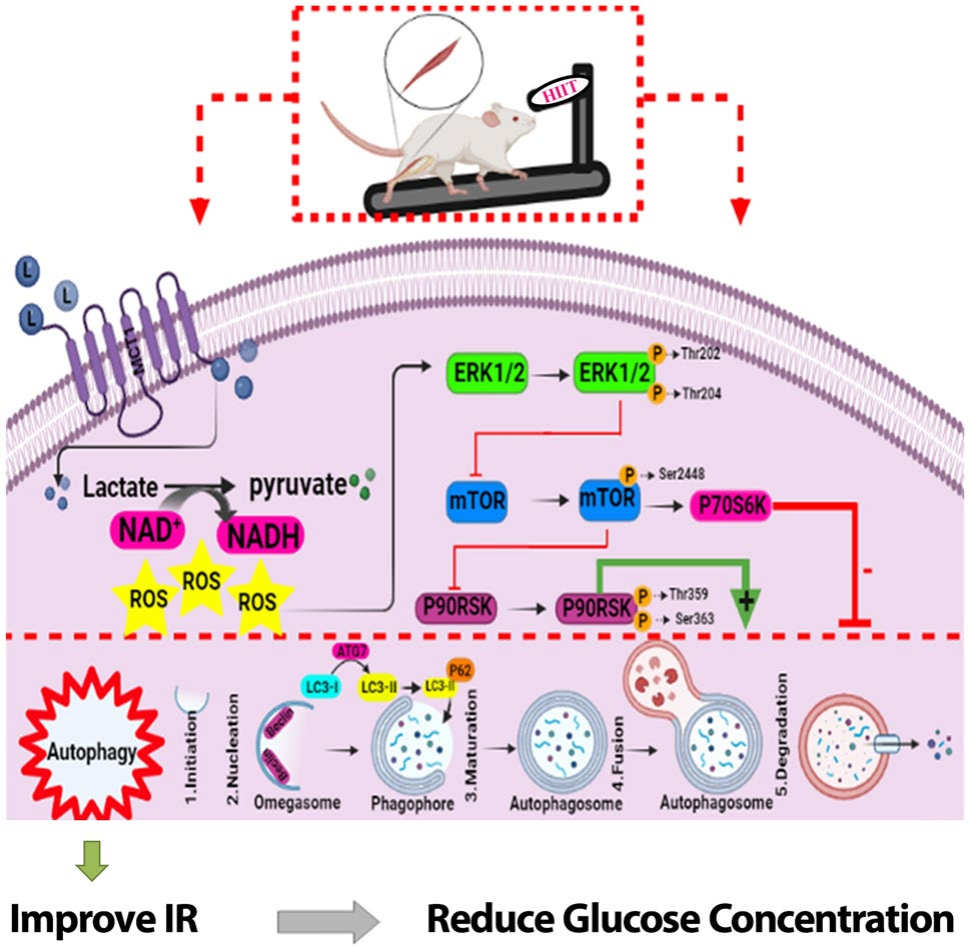
Lactate-induced autophagy activation by high-intensity interval training.

After the decrease in Beclin1, ATG7 and LC3-II levels autophagy decrease, leading to IR, which in turn increases serum levels of lactate^38,39^. IR increases glycolysis, and subsequently NADH and pyruvate while decreasing NAD+. Pyruvate is converted to lactate by lactate dehydrogenase^40^. Increasing lactate could exacerbate IR by inhibiting glucose oxidation and suppressing glucose uptake into skeletal muscles^41,42^. Lactate also increases serum glucose levels by stimulating gluconeogenesis^43^. Our results showed a significant increase in serum levels of lactate in the T2D group compared to the CO group, which is consistent with the results of a previous study^44^. Diabetes leads to a dramatic decrease in skeletal muscle MCT1^13^, which could be another reason for increased lactate levels in T2D group. After 8 weeks of HIIT, muscle MCT1 levels increased in the T2D + EX group. The authors reported that 7 weeks of endurance training increased MCT1 levels^13^. Muscular levels of MCT1 were also increased after 6 weeks of strength training in patients with T2D^45^. In our study, serum lactate levels decreased in EX group, but the decrease was not significant. It is likely that factors other than the reduction in lactate uptake play a role in hyperlactatemia^46^. It's also possible that the high serum lactate level was caused by the effects of the last training session. A significant decrease in muscle lactate levels in the diabetic compared to the healthy CO group can confirm impaired uptake of lactate by muscle. The level of muscle lactate in the T2D + EX group did not increase significantly compared to the T2D group. We believe that this is due to the increase in lactate dehydrogenase activity following exercise which brings, as a consequence, increases in pyruvate production^47^. Reducing lactate to pyruvate increase cytosolic redox potential moving muscle metabolism toward aerobic and increasing ROS is a potential result^48^. ROS have been reported to play an important role in regulating autophagy in skeletal muscles^14,49, 50^. ERK, an autophagy regulator, is upregulated by ROS^51^. Our results showed a significant increase in p-ERK in the T2D + EX group after 8 weeks of HIIT. Therefore, the cause of the increase in the p-ERK in muscle seems to be increase in muscle MCT1 levels and the uptake of lactate after eight weeks of training. The downstream target of ERK is mTOR^13^. mTOR is an autophagy inhibitor in skeletal muscle that exerts its inhibitory effects by preventing the formation of autophagosomes^52^. In the present study the p-mTOR level in the T2D group showed a significant increase compared to the CO group. After 8 weeks of HIIT, we observed a significant decrease in p-mTOR levels in the T2D + EX group. The mTOR/p70S6K signaling pathway negatively regulates autophagosome formation^53^. After decreasing p-mTOR, its downstream p70S6k is also inactivated and our results showed that after 8 weeks of training the inactive p70S6k level increased in the T2D + EX group. On the other hand, after eight weeks of HIIT, we observed an increase in the level of phosphorylated p90RSK, a downstream effector of ERK. All of the above-mentioned data confirmed increased autophagy in T2D + EX compared with T2D group. As mentioned earlier, increased autophagy could lead to improve IR. In line with this, our results showed decrees HOMA-IR and FBG in T2D + EX compared to T2D group. Therefore, we could suggest HIIT as a safe, unexpansive therapeutic approach for managing T2D (Fig. 6). However, it needs more studies especially clinical trials.

It's important to acknowledge that the absence of ROS measurements restricts the scope of our study. Moreover, by suppressing the lactate receptor or targeting specific downstream molecules within the autophagy pathway, we could enhance our ability to discern the impact of HIIT on this pathway with increased confidence.

## Conclusion

Taken together we concluded that eight weeks of high-intensity interval training could activated ERK/P90SRK while inhibiting mTOR/P70S6K signaling pathway in lactate dependent manner. It means increased autophagy which resulted in improve insulin resistance (IR) and reduce blood glucose (Supplementary Information S1).

### Supplementary Information

Supplementary Information.

## Data Availability

Data that support the findings of this study are available at Kerman University of Medical Sciences. Corresponding author should be contacted.

## References

[1] Arroyave F, Montaño D, Lizcano F (2020). Diabetes mellitus is a chronic disease that can benefit from therapy with induced pluripotent stem cells. Int. J. Mol. Sci..

[2] Khoramipour, K., Hekmatikar, A. A. & Sotvan, H. An overview of Fatmax and MFO in exercise. *Razi. J. Med. Sci*. **27**, 49–59 (2020).

[3] Larejani B, Zahedi F (2001). Epidemiology of diabetes mellitus in Iran. Iran. J. Diab. Metab..

[4] Lautsch D, Boggs R, Wang T, Gonzalez C, Milligan G, Rajpathak S (2022). Individualized HbA 1c goals, and patient awareness and attainment of goals in type 2 diabetes mellitus: A real-world multinational survey. Adv. Ther..

[5] Gonzalez CD, Lee M-S, Marchetti P, Pietropaolo M, Towns R, Vaccaro MI (2011). The emerging role of autophagy in the pathophysiology of diabetes mellitus. Autophagy.

[6] Botella J, Jamnick NA, Granata C, Genders AJ, Perri E, Jabar T (2022). Exercise and training regulation of autophagy markers in human and rat skeletal muscle. Int. J. Mol. Sci..

[7] Feng Y, He D, Yao Z, Klionsky DJ (2014). The machinery of macroautophagy. Cell Res..

[8] Barlow AD, Thomas DC (2015). Autophagy in diabetes: β-cell dysfunction, insulin resistance, and complications. DNA Cell Biol..

[9] Munasinghe PE, Riu F, Dixit P, Edamatsu M, Saxena P, Hamer NS (2016). Type-2 diabetes increases autophagy in the human heart through promotion of Beclin-1 mediated pathway. Int. J. Cardiol..

[10] Møller AB, Kampmann U, Hedegaard J, Thorsen K, Nordentoft I, Vendelbo MH (2017). Altered gene expression and repressed markers of autophagy in skeletal muscle of insulin resistant patients with type 2 diabetes. Sci. Rep..

[11] Cho DK, Choi DH, Cho JY (2017). Effect of treadmill exercise on skeletal muscle autophagy in rats with obesity induced by a high-fat diet. J. Exerc. Nutr. Biochem..

[12] Choi JW, Ohn JH, Jung HS, Park YJ, Jang HC, Chung SS (2018). Carnitine induces autophagy and restores high-fat diet-induced mitochondrial dysfunction. Metabolism..

[13] Nikooie R, Moflehi D, Zand S (2021). Lactate regulates autophagy through ROS-mediated activation of ERK1/2/m-TOR/p-70S6K pathway in skeletal muscle. J. Cell Commun. Signal..

[14] Rahman M, Mofarrahi M, Kristof AS, Nkengfac B, Harel S, Hussain SN (2014). Reactive oxygen species regulation of autophagy in skeletal muscles. Antioxid. Redox Signal..

[15] Brooks GA, Osmond AD, Arevalo JA, Duong JJ, Curl CC, Moreno-Santillan DD (2023). Lactate as a myokine and exerkine: drivers and signals of physiology and metabolism. J. Appl. Physiol..

[16] McCarthy SF, Bornath DP, Jarosz C, Tucker JA, Medeiros PJ, Kenno KA (2023). Intense interval exercise induces lactate accumulation and a greater suppression of acylated ghrelin compared to submaximal exercise in middle-aged adults. J. Appl. Physiol..

[17] Khoramipour K, Bejeshk MA, Rajizadeh MA, Najafipour H, Dehghan P, Farahmand F (2023). High-intensity interval training ameliorates molecular changes in the hippocampus of male rats with the diabetic brain: The role of adiponectin. Mol. Neurobiol..

[18] Khajehlandi, M. *et al.* Endurance training regulates expression of some angiogenesis-related genes in cardiac tissue of experimentally induced diabetic rats. *Biomolecules* **11**(4), 498 (2021). 10.3390/biom11040498 PMC8066303 33806202

[19] Orumiyehei A, Khoramipour K, Rezaei MH, Madadizadeh E, Meymandi MS, Mohammadi F (2022). High-intensity interval training-induced hippocampal molecular changes associated with improvement in anxiety-like behavior but not cognitive function in rats with type 2 diabetes. Brain Sci..

[20] Khoramipour, K. *et al.* High intensity interval training can ameliorate hypothalamic appetite regulation in male rats with Type 2 diabetes: The role of leptin. *Cell. Mol. Neurobiol.* **12**, 1–3 (2023). 10.1007/s10571-023-01421-w PMC11407713 37828299

[21] Rezaei, M. H. *et al.* Leptin signaling could mediate hippocampal decumulation of beta-amyloid and tau induced by high-intensity interval training in rats with type 2 diabetes. *Cell. Mol. Neurobiol.* **43**(7), 3465–3478 (2023). 10.1007/s10571-023-01357-1 PMC11409991 37378849

[22] Rajizadeh, M. A. *et al.* Adiponectin receptor 1 could explain the sex differences in molecular basis of cognitive improvements induced by exercise training in type 2 diabetic rats. *Sci. Rep.* **13**(1), 16267 (2023). 10.1038/s41598-023-43519-7 PMC10533546 37758935

[23] Ebrahimnezhad, N., Nayebifar, S., Soltani, Z. & Khoramipour, K. High-intensity interval training reduced oxidative stress and apoptosis in the hippocampus of male rats with type 2 diabetes: The role of the PGC1α-Keap1-Nrf2 signaling pathway. *Iran. J. Basic. Med. Sci.* **26**(11), 1313 (2023). 10.22038/IJBMS.2023.70833.15387 PMC10598812 37885999

[24] Basereh, A., Ebrahim, K., Hovanloo, F., Dehghan, P. & Khoramipour, K. Effect of blood flow restriction deal during isometric exercise on growth hormone and testosterone active males. *Sport. Psychol.* **9**(33), 51–68 (2017).

[25] Rahmaty, S., Dehghan, P., Khoramipour, K. & Saboory, M. The effect of listening to brain waves’ relaxing and exciting music during intense endurance training on blood cortisol levels of adult men. *Am. J. Sports Sci. Med.* **3**(4), 77–81 (2015).

[26] Sims-Robinson C, Kim B, Rosko A, Feldman EL (2010). How does diabetes accelerate Alzheimer disease pathology?. Nat. Rev. Neurol..

[27] DeFronzo RA, Tripathy D (2009). Skeletal muscle insulin resistance is the primary defect in type 2 diabetes. Diabetes Care..

[28] Neel BA, Lin Y, Pessin JE (2013). Skeletal muscle autophagy: A new metabolic regulator. Trends Endocrinol. Metab. TEM..

[29] Lu L, Ma Y, Deng J, Xie J, Huang C (2022). Lower ATG7 levels are associated with a higher risk of gestational diabetes mellitus: A cross-sectional study. Diab. Metab. Syndr. Obes. Targets Ther..

[30] Yang L, Li P, Fu S, Calay ES, Hotamisligil GS (2010). Defective hepatic autophagy in obesity promotes ER stress and causes insulin resistance. Cell Metab..

[31] Feng Z, Bai L, Yan J, Li Y, Shen W, Wang Y (2011). Mitochondrial dynamic remodeling in strenuous exercise-induced muscle and mitochondrial dysfunction: Regulatory effects of hydroxytyrosol. Free Rad. Biol. Med..

[32] Luo L, Lu A-M, Wang Y, Hong A, Chen Y, Hu J (2013). Chronic resistance training activates autophagy and reduces apoptosis of muscle cells by modulating IGF-1 and its receptors, Akt/mTOR and Akt/FOXO3a signaling in aged rats. Exp. Gerontol..

[33] Chul Jang Y, Hwang DJ, Koo JH, Um HS, Lee NH, Yeom DC (2018). Association of exercise-induced autophagy upregulation and apoptosis suppression with neuroprotection against pharmacologically induced Parkinson's disease. J. Exerc. Nutr. Biochem..

[34] Yang B, Sun J, Yuan Y, Sun Z (2018). Effects of atorvastatin on autophagy in skeletal muscles of diabetic rats. J. Diab. Investig..

[35] Liu H-Y, Han J, Cao SY, Hong T, Zhuo D, Shi J (2009). Hepatic autophagy is suppressed in the presence of insulin resistance and hyperinsulinemia: Inhibition of FoxO1-dependent expression of key autophagy genes by insulin. J. Biol. Chem..

[36] Jamart C, Francaux M, Millet GY, Deldicque L, Frère D, Féasson L (2012). Modulation of autophagy and ubiquitin-proteasome pathways during ultra-endurance running. J. Appl. Physiol..

[37] Brandt N, Gunnarsson TP, Bangsbo J, Pilegaard H (2018). Exercise and exercise training-induced increase in autophagy markers in human skeletal muscle. Physiol. Rep..

[38] Wu Y, Dong Y, Atefi M, Liu Y, Elshimali Y, Vadgama JV (2016). Lactate, a neglected factor for diabetes and cancer interaction. Mediat. Inflamm..

[39] Zhang K (2018). "NO" to autophagy: Fat does the trick for diabetes. Diabetes..

[40] Simoneau JA, Colberg SR, Thaete FL, Kelley DE (1995). Skeletal muscle glycolytic and oxidative enzyme capacities are determinants of insulin sensitivity and muscle composition in obese women. FASEB J..

[41] Hamamdžić M, Hrabač B, Alić A, Pašić-Juhas E, Hodžić A (2008). Effect of lactate on insulin action in rats. Bosnian J. Basic Med. Sci..

[42] Choi CS, Kim YB, Lee FN, Zabolotny JM, Kahn BB, Youn JH (2002). Lactate induces insulin resistance in skeletal muscle by suppressing glycolysis and impairing insulin signaling. Am. J. Physiol. Endocrinol. Metab..

[43] Metz L, Sirvent P, Py G, Brun J-F, Fédou C, Raynaud E (2005). Relationship between blood lactate concentration and substrate utilization during exercise in type 2 diabetic postmenopausal women. Metabolism..

[44] Ishitobi M, Hosaka T, Morita N, Kondo K, Murashima T, Kitahara A (2019). Serum lactate levels are associated with serum alanine aminotransferase and total bilirubin levels in patients with type 2 diabetes mellitus: A cross-sectional study. Diab. Res. Clin. Pract..

[45] Juel C, Holten MK, Dela F (2004). Effects of strength training on muscle lactate release and MCT1 and MCT4 content in healthy and type 2 diabetic humans. J. Physiol..

[46] DiGirolamo M, Newby FD, Lovejoy J (1992). Lactate production in adipose tissue: A regulated function with extra-adipose implications. FASEB J..

[47] Carnevali L, Eder R, Lira FSD, Lima WP, Gonçalves DC, Zanchi NE (2012). Effects of high-intensity intermittent training on carnitine palmitoyl transferase activity in the gastrocnemius muscle of rats. Braz. J. Med. Biol. Res..

[48] Nalbandian M, Takeda M (2016). Lactate as a signaling molecule that regulates exercise-induced adaptations. Biology..

[49] Dobrowolny G, Aucello M, Rizzuto E, Beccafico S, Mammucari C, Bonconpagni S (2008). Skeletal muscle is a primary target of SOD1G93A-mediated toxicity. Cell Metab..

[50] Rodney GG, Pal R, Abo-Zahrah R (2016). Redox regulation of autophagy in skeletal muscle. Free Rad. Biol. Med..

[51] Wu H-W, Li H-F, Wu X-Y, Zhao J, Guo J (2008). Reactive oxygen species mediate ERK activation through different Raf-1-dependent signaling pathways following cerebral ischemia. Neurosci. Lett..

[52] Risson V, Mazelin L, Roceri M, Sanchez H, Moncollin V, Corneloup C (2009). Muscle inactivation of mTOR causes metabolic and dystrophin defects leading to severe myopathy. J. Cell Biol..

[53] Saiki S, Sasazawa Y, Imamichi Y, Kawajiri S, Fujimaki T, Tanida I (2011). Caffeine induces apoptosis by enhancement of autophagy via PI3K/Akt/mTOR/p70S6K inhibition. Autophagy..

