# Author Correction: Lactate-induced autophagy activation: unraveling the therapeutic impact of high-intensity interval training on insulin resistance in type 2 diabetic rats

**DOI:** 10.1038/s41598-024-53618-8

**Published:** 2024-02-06

**Authors:** Hossein Pirani, Afsaneh Soltany, Maryam Hossein Rezaei, Adeleh Khodabakhshi Fard, Rohollah Nikooie, Kimya Khoramipoor, Karim Chamari, Kayvan Khoramipour

**Affiliations:** 1 Department of Basic Sciences, Chabahar Maritime University, Chabahar, Iran; 2 Department of Biology, Faculty of Science, University of Shiraz, Shiraz, Iran; 3 Department of Exercise Physiology, Faculty of Physical Education and Sport Sciences, Shahid Bahonar University, Kerman, Iran; 4 Department on Nutrition, Faculty of Public Health, Kerman University of Medical Sciences, Kerman, Iran; 5 Neuroscience Research Center, Institute of Neuropharmacology, Kerman University of Medical Sciences, Kerman, Iran; 6 Department of Nursing, Faculty of Nursing and Midwifery, Kurdistan University of Medical Sciences, Kurdistan, Iran; 7 Higher Institute of Sport and Physical Education, ISSEP Ksar Said, Manouba University, Manouba, Tunisia; 8 Neuroscience Research Center, Institute of Neuropharmacology and Department of Physiology and Pharmacology, Afzalipour School of Medicine, Kerman University of Medical Sciences, Kerman, Iran

Correction to: *Scientific Reports* 10.1038/s41598-023-50589-0, published online 11 January 2024

The original version of this Article contained an error in the spelling of the author Rohollah Nikooie which was incorrectly given as Rohollah Nikooei.

The original Article has been corrected.

