# Supplementary material for: Lactate-induced autophagy activation: unraveling the therapeutic impact of high-intensity interval training on insulin resistance in type 2 diabetic rats
Source: Sci Rep. 2024 Jan 11;14:1108. doi: 10.1038/s41598-023-50589-0 (PMC10784291; doi:10.1038/s41598-023-50589-0)
Supplement: Supplementary file 1 — Supplementary Information. [file 41598_2023_50589_MOESM1_ESM.docx]

**Used in manuscript**

Figure S1. **MCT1: lactate transporter monocarboxylate transporter**  for Figure 3. Note. all blot membranes were cut into stripes prior to hybridization with antibodies. Membrane edges are not clear in some cases, due to high signal-to-noise ratio of luminescence intensity

**Used in manuscript**

Figure S2. **P- ERK1/2: phosphorylated mitogen-activated protein kinases (MAPKs) ½** for Figure 5 (A,B),  Note. all blot membranes were cut into stripes prior to hybridization with antibodies. Membrane edges are not clear in some cases, due to high signal-to-noise ratio of luminescence intensity

**Used in manuscript**

Figure S3. **p- mTOR: mammalian target of rapamycin** for Figure 5. (C) Note. all blot membranes were cut into stripes prior to hybridization with antibodies. Membrane edges are not clear in some cases, due to high signal-to-noise ratio of luminescence intensity

**Used in manuscript**

Figure S4 . **p70S6k: Ribosomal protein S6 kinase beta-1** for Figure 5. (D) Note. all blot membranes were cut into stripes prior to hybridization with antibodies. Membrane edges are not clear in some cases, due to high signal-to-noise ratio of luminescence intensity

**Used in manuscript**

Figure S5. **P-90RSK: p90 ribosomal S6 kinases** for Figure 5. (E) Note. all blot membranes were cut into stripes prior to hybridization with antibodies. Membrane edges are not clear in some cases, due to high signal-to-noise ratio of luminescence intensity

**Used in manuscript**

Figure S6. **ATG7: autophagy related** for Figure 5. (F) Note. all blot membranes were cut into stripes prior to hybridization with antibodies. Membrane edges are not clear in some cases, due to high signal-to-noise ratio of luminescence intensity

**Used in manuscript**

Figure S7. **Beclin-1** for Figure 5. (G) Note. all blot membranes were cut into stripes prior to hybridization with antibodies. Membrane edges are not clear in some cases, due to high signal-to-noise ratio of luminescence intensity

**Used in manuscript**

Figure S8.  **LC3-I** for Figure 5. (H) Note. all blot membranes were cut into stripes prior to hybridization with antibodies. Membrane edges are not clear in some cases, due to high signal-to-noise ratio of luminescence intensity

**Used in manuscript**

Figure S8.  **LC3-II** for Figure 5. (I) Note. all blot membranes were cut into stripes prior to hybridization with antibodies. Membrane edges are not clear in some cases, due to high signal-to-noise ratio of luminescence intensity

**Used in manuscript**

Figure S9. **P62** for Figure 5. (K) Note. all blot membranes were cut into stripes prior to hybridization with antibodies. Membrane edges are not clear in some cases, due to high signal-to-noise ratio of luminescence intensity

**B-actin**

Note. all blot membranes were cut into stripes prior to hybridization with antibodies. Membrane edges are not clear in some cases, due to high signal-to-noise ratio of luminescence intensity
